# Clinical features most frequently present in patients with concomitant diabetic kidney disease and diabetic retinopathy

**DOI:** 10.20945/2359-4292-2023-0377

**Published:** 2024-07-01

**Authors:** Mateus Augusto dos Reis, Janine Alessi, Josiane Schneiders, Clara Krummenauer Maraschin, Gabriela Oliveira Gonçalves Molino, Bianca Gomes Correa, Daniel Lavinsky, Gabriela Heiden Teló, Beatriz D. Schaan

**Affiliations:** 1 Programa de Pós-graduação em Ciências Médicas Universidade Federal do Rio Grande do Sul Porto Alegre RS Brasil Programa de Pós-graduação em Ciências Médicas – Endocrinologia, Universidade Federal do Rio Grande do Sul, Porto Alegre, RS, Brasil; 2 Universidade Feevale Novo Hamburgo RS Brasil Universidade Feevale, Novo Hamburgo, RS, Brasil; 3 Universidade Federal do Rio Grande do Sul Porto Alegre RS Brasil Universidade Federal do Rio Grande do Sul, Porto Alegre, RS, Brasil; 4 Divisão de Endocrinologia Hospital de Clínicas de Porto Alegre Porto Alegre RS Brasil Divisão de Endocrinologia, Hospital de Clínicas de Porto Alegre, Porto Alegre, RS, Brasil; 5 Faculdade de Medicina Programa de Pós-graduação em Medicina e Ciências da Saúde Pontifícia Universidade Católica do Rio Grande do Sul Porto Alegre RS Brasil Faculdade de Medicina, Programa de Pós-graduação em Medicina e Ciências da Saúde, Pontifícia Universidade Católica do Rio Grande do Sul, Porto Alegre, RS, Brasil; 6 IATS CNPq Porto Alegre RS Brasil Instituto Nacional de Ciência e Tecnologia para Avaliação de Tecnologias em Saúde (IATS) – CNPq, Porto Alegre, RS, Brasil

**Keywords:** Diabetic retinopathy, diabetic kidney disease, diabetes mellitus, diabetic complications

## Abstract

**Objective:**

To evaluate the profile of patients with diabetic kidney disease (DKD) with and without concomitant diabetic retinopathy (DR) to identify clinical predictors of the development of both complications together.

**Subjects and methods:**

Cross-sectional study including patients with type 1 and type 2 diabetes and DKD followed at the endocrinology division of a public hospital in Southern Brazil and referred for retinography assessment. The definition of DR was the occurrence of any diabetes-related damage identified in color fundus photographs under mydriasis. Urinary albumin excretion ≥ 14 mg/L and/or glomerular filtration rate < 60 mL/min/1.73 m^2^ (CKD-EPI equation) were used to define DKD. Factors evaluated included the clinical differences of the participants according to the presence or absence of DR. Multiple regression models were used to identify predictors of DR presence according to the clinical characteristics evaluated.

**Results:**

The study included 517 patients with DKD, 433 (83.7%) of whom had type 2 diabetes (median age 64.7 years [interquartile range 59-73 years] years, 59.8% women, 83.4% white) and 84 (16.3%) had type 1 diabetes (median age 46.6 years [interquartile range 33.5-54.2 years], 46.4% women, 91.7% white). Patients with type 2 diabetes and DR (versus those without DR) were more often on insulin (odds ratio [OR] 3.63, 95% confidence interval [CI] 1.89-7.00), had diabetes for longer (OR 1.04, 95% CI 1.02-1.07), and had higher systolic blood pressure (OR 1.01, 95% CI 1.00-1.02). No predictors of DR presence were identified in participants with type 1 diabetes.

**Conclusion:**

Among patients with DKD and type 2 diabetes, insulin use, longer diabetes duration, and higher systolic blood pressure level were associated with the presence of DR.

## INTRODUCTION

Diabetes mellitus is a metabolic disease that may lead to chronic microvascular and macrovascular complications (1). Diabetic retinopathy (DR) and diabetic kidney disease (DKD) are microvascular complications that may manifest independently or concurrently, contributing to increased morbidity, mortality, and medical costs and reduced quality of life (1-3). Both complications remain highly prevalent worldwide. Indeed, DR affects about 22% of the patients with diabetes and is one of the most common causes of preventable blindness in the economically active population, while DKD affects between 21%-83% of individuals with diabetes and is the main cause of end-stage renal disease in most developed countries (4-6).

Despite being independent entities, DKD and DR are often present in association, as they may share common pathogenetic mechanisms (7). Corroborating this premise, studies have shown that DKD markers, such as increased albuminuria and reduced glomerular filtration rate (GFR), are predictors of DR (9,10), and albuminuria remission seems to have a protective effect in DR development (11). Furthermore, baseline DR severity may constitute a prognostic factor for future DKD progression, especially in patients with type 2 diabetes (10).

Therefore, the identification of clinical predictors that could discern which patients with DKD are at greater risk of developing DR may support early and aggressive interventions to prevent this outcome. Based on these considerations, the aim of this study was to compare the characteristics of patients with DKD with and without DR to identify clinical predictors of developing both complications (DKD and DR) together.

## SUBJECTS AND METHODS

### Design and participants

This was a cross-sectional study in patients with DKD due to type 1 or 2 diabetes who were followed at the endocrinology division of a public hospital in Southern Brazil and were referred for retinography assessment. Clinical and demographic data were collected by interviews and extracted from medical records between 2019 and 2021. The inclusion criteria included patients of both sexes, aged ≥ 18 years, nonpregnant, and diagnosed with type 1 or type 2 diabetes according to American Diabetes Association criteria (12). Participants whose results of color fundus photographs (CFPs) were impossible to classify and those without serum creatinine and urinary albumin concentration (UAC) measurements available in medical records were excluded.

The study was reported according to the Strengthening the Reporting of Observational Studies in Epidemiology (STROBE) guideline (13).

### Ethical aspects

The study was carried out in accordance with the Declaration of Helsinki 2004 and followed all relevant guidelines and regulations. The study protocol was approved by the Research Ethics Committee of *Hospital das Clínicas de Porto Alegre* (2019-0113, CAAE 07744819.9.0000.5327). All authors signed a confidentiality document for data use, and all participants signed an informed consent form.

### Study procedures

Patients who attended the main institution for CFP examination from June 2019 to March 2020 were invited to participate. An interview was performed to gather clinical data (age, skin color, smoking habit, age at diagnosis of diabetes) and information about other diagnoses and medication use (antidiabetics, insulin, antihypertensives, lipid-lowering drugs). Subsequently, blood and urinary samples were collected for laboratory tests (creatinine, urea, glucose, glycated hemoglobin [HbA1c], total cholesterol, HDL cholesterol, triglycerides, and UAC) if the patient had not performed these laboratory tests in the same institutional laboratory in the previous year. Data on weight and height (for body mass index calculation), as well as blood pressure, were collected from the electronic medical records of the health appointment in which the CFP was requested. Information about the presence of cardiovascular disease was obtained from medical records. Cardiovascular disease was defined as the presence of previous cardiovascular events, including coronary heart disease, stroke, or heart failure. Hypertension was defined as systolic blood pressure (SBP) ≥ 140 mmHg and/or diastolic blood pressure (DBP) ≥ 90 mmHg and/or use of antihypertensive treatment.

To achieve adequate power for the proposed analyses, a second round of participants were enrolled. At this point, patients who had a CFP carried out between January 2021 and November 2021 were included. These patients also had their clinical information collected from the electronic medical record of the medical appointment closest to the CFP.

Levels of HbA1c were measured using the high-performance liquid chromatography method, as employed by the Diabetes Control and Complications Trial (DCCT) (14). Levels of total cholesterol, HDL cholesterol, and triglycerides were determined using a standard analytical method, and LDL cholesterol was calculated using the Friedewald formula (15). All samples for laboratory tests were collected in the same institutional laboratory, and the results were recorded by study researchers in an electronic database.

A trained technician obtained retinal images to evaluate the presence of DR. The examination was performed under mydriasis, and two images of the posterior segment of each patient’s eye were obtained (one centered on the macula and the other centered on the optic nerve). A retina specialist interpreted the results. Retinal images were obtained using a Canon CR-2 retinal camera equipped with a Canon EOS 40D digital camera (Canon Inc., Tokyo, Japan). The CFP results were classified according to the International Council of Ophthalmology Diabetic Retinopathy (ICDR), and the result of the worst eye was considered (16).

The presence of DKD was assessed with measurement of UAC and calculation of the estimated GFR using the Chronic Kidney Disease Epidemiology Collaboration (CKD-EPI) equation (17). The occurrence of DKD was defined as a GFR < 60 mL/min/1.73 m^2^ and/or UAC from a single urine sample ≥ 14 mg/L (18-20).

### Study outcome

The primary outcome was the clinical characteristics of the participants with DKD categorized by the presence or absence of DR and type of diabetes.

### Statistical analysis

Demographics and clinical data are presented as mean ± standard deviation for variables exhibiting normal distribution and median (interquartile range [IQR]), frequencies, and percentages for those demonstrating asymmetric distribution. For the analysis, patients with DKD were stratified according to the presence or absence of DR. The groups were compared using the chi-square test in the case of categorical variables and the Mann-Whitney test in the case of continuous variables. A multivariable logistic regression model was designed to assess the association between the participants’ clinical characteristics and DR presence, controlling for potential confounders. The logistic regression model incorporated the variables “duration of diabetes since diagnosis,” “SBP,” “insulin use,” “HbA1c value,” and “GFR.” The first model was designed for type 1 diabetes, and the second for type 2 diabetes. The data are presented as odds ratios (ORs) and 95% confidence intervals (CIs). P levels ≤ 0.05 were considered statistically significant.

The statistical analysis was performed using the software SPSS, Version 20.0 (IBM Corp., Armonk, NY, USA).

## RESULTS

**Figure 1:**
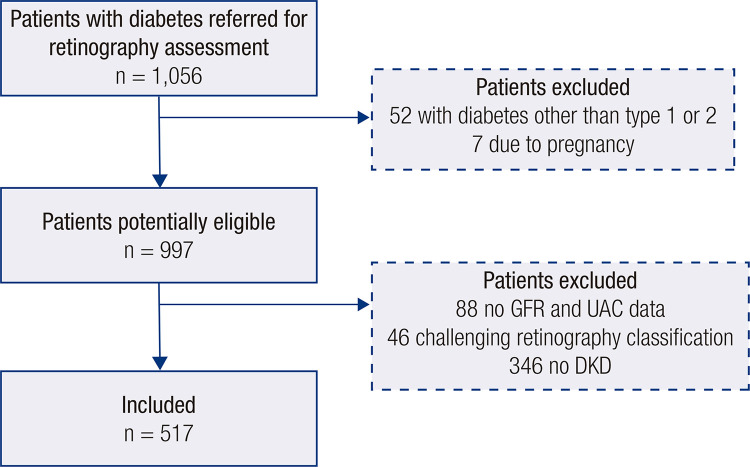
Flow diagram of the study. Abbreviations: DKD, diabetes kidney disease; GFR, glomerular filtration rate; UAC, urinary albumin concentration (obtained from a single urine sample).

Among 997 potentially eligible participants identified (Figure 1), 517 with DKD were included. Of these, 433 (83.8%) had type 2 diabetes and 236 (45.6%) had DR. Among the patients with DR, 81 (34.3%) had mild nonproliferative DR, 84 (35.6%) had moderate nonproliferative DR, 16 (6.8%) had severe nonproliferative DR, and 55 (23.3%) had proliferative DR. Macular edema was identified in 34 (3.8%) participants. In 37 (4.1%) participants, the CFP alterations found were impossible to classify.

Patients with type 1 diabetes (n = 84, 16.2%) had a median age of 44.6 years (33.5-54.2 years); 46.4% were women and 91.7% were white. The median duration of diabetes since diagnosis was 29.1 years (21.0-36.5 years), and the median HbA1c level was 9.2% (8.0-10.6%) or 77.0 mmol/mol (64.0-92.0 mmol/mol). Most patients with type 1 diabetes (n = 48, 57.1%) had DR (Table 1).

**Table 1:** Baseline characteristics of the patients with type 1 diabetes mellitus and diabetic kidney disease

	Without DR (n = 36)	With DR (n = 48)	P values
Age (years)	32.8 (21-40.7)	43.4 (35.2-50.7)	<0.01
Sex (% women)	21 (58.3)	18 (37.5)	0.06
Skin color (% white)	34 (94.4)	43 (89.6)	0.42
BMI (kg/m^2^)	24.6 (22.3-26.6)	26.5 (22.6-30.1)	0.26
Diabetes duration (years)	16.1 (8.2-19)	24.5 (17-33)	<0.01
HbA1c (%) / (mmol/mol)	9.8 (8.2-11) /84.0 (66.0-97.0)	9.3 (8.2-10.2) /78.0 (66.0-88.0)	0.28
Systolic blood pressure (mmHg)	116.5 (102-124.5)	128.4 (110-140)	0.03
Diastolic blood pressure (mmHg)	71 (60-80)	86.5 (70-90)	0.08
UAC (mg/L)	134.6 (17-51.5)	291.9 (20-190)	0.14
GFR (mL/min/1.73 m^2^)	103.8 (98.7-118.7)	78 (47-107.7)	<0.01
Total cholesterol (mg/dL)	184.4 (150-200)	171.4 (141.5-191.5)	0.49
HDL cholesterol (mg/dL)	46.7 (35-54)	48.2 (38.5-54)	0.66
Triglycerides (mg/dL)	253.6 (95-209.5)	136.2 (70.7-143)	0.07
Smoking	1 (2.8)	7 (14.6)	0.07
Cardiovascular disease	3 (8.3)	8 (17)	0.24
Use of ACE inhibitors	13 (36.1)	19 (39.6)	0.74
Use of antihypertensives	15 (41.7)	27 (56.2)	0.18
Use of statins	8 (22.2)	17 (35.4)	0.19

Data are shown as median (interquartile range) or n (%). P values ≤ 0.05 indicate a significant difference. Abbreviations: ACE, angiotensin-converting enzyme; BMI, body mass index; DKD, diabetic kidney disease; DR, diabetic retinopathy; GFR, glomerular filtration rate; HbA1c, glycated hemoglobin; HDL cholesterol, high-density lipoprotein cholesterol; UAC: urinary albumin concentration (obtained from a single urine sample).

Among patients with type 2 diabetes (n = 433, 83.8%), the median age was 64.7 years (59.0-73.0 years), 59.8% were women, and 83.4% were white. The median duration of diabetes since diagnosis was 18.6 years (11-25.5 years), and the mean HbA1c level was 8.9% (6.9-10.0%) or 74.0 mmol/mol (52.0-86.0 mmol/mol). Only a minority of patients with type 2 diabetes had DR (188, 43.4%) (Table 2).

**Table 2:** Baseline characteristics of patients with type 2 diabetes mellitus and diabetic kidney disease

	Without DR (n = 245)	With DR (n = 188)	P values
Age (years)	60.4 (53 – 69)	61.1 (54-68)	0.98
Sex (% women)	147 (60)	112 (59.6)	0.93
Skin color (% white)	203 (82.9)	158 (84)	0.74
BMI (kg/m^2^)	33.4 (28.1-37.2)	32 (27.3-35.5)	0.06
Diabetes duration (years)	13.8 (6-19)	18.1 (11-23)	<0.01
HbA1c (%) / (mmol/mol)	8.6 (7.3-9.6) / 70.0 (56.0-81.0)	9.2 (8-10.1) / 77.0 (64.0-87.0)	<0.01
Systolic blood pressure (mmHg)	132.7 (120-140)	139.2 (121.2-150.7)	<0.01
Diastolic blood pressure (mmHg)	78.3 (70-86)	79.8 (70-90)	0.47
Hypertension (%)	207 (84.5)	165 (87.8)	0.33
UAC (mg/L)	219.3 (18-136)	349.9 (26-280)	<0.01
GFR (mL/min/1.73 m^2^)	73.6 (48-97)	67.2 (45-89)	0.05
Total cholesterol (mg/dL)	179.9 (142-205.5)	185.3 (152-207)	0.29
HDL cholesterol (mg/dL)	45.4 (34-53)	46.1 (35.7-52.2)	0.37
Triglycerides (mg/dL)	209.9 (95.2-210.7)	296.5 (108-216)	0.34
Smoking	25 (10.2)	18 (9.6)	0.83
Cardiovascular disease	80 (32.9)	65 (34.8)	0.69
Use of ACE inhibitors	139 (56.7)	118 (62.8)	0.20
Use of antihypertensives	211 (86.1)	165 (87.8)	0.61
Use of statins	168 (68.6)	133 (70.7)	0.62
Use of metformin	198 (80.8)	139 (73.9)	0.09
Use of sulfonylurea	70 (28.6)	41 (21.8)	0.11
Use of iSGLT2	32 (13.1)	21 (11.2)	0.55
Use of insulin	164 (66.9)	173 (92.0)	<0.01

Data are shown as median (interquartile range) or n (%). P values ≤ 0.05 indicate a significant difference. Abbreviations: ACE, angiotensin-converting enzyme; BMI, body mass index; DKD, diabetic kidney disease; DR, diabetic retinopathy; GFR, glomerular filtration rate; HbA1c, glycated hemoglobin; HDL cholesterol, high-density lipoprotein cholesterol; iSGLT2, sodium-glucose cotransporter type 2 inhibitors; UAC: urinary albumin concentration (obtained from a single urine sample).

Table 3 shows the results of the logistic regression analysis evaluating the association between the participants’ clinical characteristics and the presence of DR in patients with type 1 diabetes. The assessed associations were not significant.

**Table 3:** Logistic regression analysis evaluating the association between the participants’ clinical characteristics and the presence of diabetic retinopathy in patients with type 1 diabetes

Variables	Presence of DR	P
Age	1.00 (0.95-1.06)	0.85
Diabetes duration	0.95 (0.89-1.02)	0.20
Systolic blood pressure	0.99 (0.96-1.02)	0.74
GFR	1.01 (1.00-1.03)	0.06

Abbreviation: DR, diabetic retinopathy; GFR, glomerular filtration rate.

Table 4 shows the results of the logistic regression analysis evaluating the association between the participants’ clinical characteristics and the presence of DR in patients with type 2 diabetes. Patients with DR, relative to those without DR, were more frequently on insulin (OR 3.63, 95% CI 1.89-7.00), had a longer duration of diabetes since diagnosis (OR 1.04, 95% CI 1.02-1.07), and had higher SBP level (OR 1.01, 95% CI 1.00-1.02).

**Table 4:** Logistic regression analysis evaluating the association between the participants’ clinical characteristics and the presence of diabetic retinopathy in patients with type 2 diabetes

Variables	Presence of DR	P
Insulin use	3.63 (1.89-7.00)	<0.01
Diabetes duration	1.04 (1.02-1.07)	<0.01
Systolic blood pressure	1.01 (1.00-1.02)	<0.01
GFR	1.00 (0.99-1.01)	0.62
HbA1c	1.09 (0.97-1.23)	0.14
UAC	1.00 (1.00-1.00)	0.08

Abbreviations: GFR, glomerular filtration rate; HbA1c, glycated hemoglobin; UAC, urinary albumin concentration (obtained from a single urine sample).

## DISCUSSION

The present study, including 517 patients with DKD, found associations between some clinical features and the presence of DR in patients with type 2 diabetes. These clinical features included insulin use, longer diabetes duration since diagnosis, and higher SBP levels. These findings are relevant as they indicate that, because both complications (DR and DKD) share common pathogenetic mechanisms (21), some patients with DKD are at increased risk of presenting DR associated with DKD. This evidence may help guide strategies to optimize DR screening in patients with DKD. In contrast, the present study found no characteristics associated with the presence of DR in patients with type 1 diabetes and DKD.

The identification of clinical features associated with a higher risk of development of DR enables the detection of patients with DKD who require closer monitoring for the potential for the development of DR. The prevalence of DR in patients with diabetes globally is around 22.3% (4), but the present study found a substantially higher prevalence (45.6%). This may have resulted from the selected patients with DKD having a possible higher risk of DR development. Corroborating this hypothesis, Pavkov et al. have demonstrated that the DKD prevalence is higher in patients with DR and reported a DKD prevalence of 23.4% in patients without DR and 36.2% in those with DR (22). Additionally, the patients included in the present study may have had diabetes of more difficult control and, consequently, more prone to the development of chronic complications. This hypothesis is in line with other characteristics of the participants included in the present study, such as the high frequency of insulin use among patients with type 2 diabetes. Moreover, the study was conducted in a tertiary care public hospital, where the most severe cases of diabetes are treated. This factor, along with the high rate of patients with type 2 diabetes using insulin and the longer duration and severity of diabetes, contribute to the high prevalence of DKD observed in the cohort.

Regarding the clinical predictors included in the analysis, the duration of diabetes is an established predictor for the development of DR and other microvascular complications in patients with type 2 diabetes (23-25). This evidence is aligned with the finding that the patients with DR had longer diabetes duration and possibly more advanced disease. Previous studies have also demonstrated that SBP levels are higher in patients with concomitant DKD and DR and contribute not only to the emergence of DR but also to its progression (26,27).

The results of the present study showed a trend towards lower GFR and higher UAC in patients with DKD and DR, which were no longer present after correction for confounders in the multivariable analysis. However, previous studies in patients with type 2 diabetes have shown that DR is an independent predictor of worsening renal function (28). In addition, studies suggest that the DR severity at baseline is associated with a faster decline in renal function and progression of albuminuria, with a risk of DKD progression 2.9 times higher in patients with nonproliferative DR and 16.6 times higher in patients with proliferative DR (29).

For type 1 diabetes, the present study found no association between clinical predictors and the presence of DR. This may have resulted from the small number of patients with this type of diabetes in the study sample. A Brazilian multicenter study including 1,760 patients with type 1 diabetes showed that a longer duration of diabetes and high levels of HbA1c are associated with the development of DR. However, the study included patients with and without DKD, which differs from the present study (30). Another analysis from this same cohort showed that female sex, longer duration of diabetes, low economic status, and high levels of HbA1c, uric acid, and SBP were associated with DKD (31).

The present study used CFPs as a diagnostic method for DR, but another, more sensitive imaging modality, optical coherence tomography (OCT), has recently been used for DR evaluation. A type of fundus examination, OCT obtains information on retinal layers using tomographic scans that can detect early changes in vascular and retinal morphology, either by measuring inner retinal thickness or choroidal thickness changes, making it an important tool for the management and monitoring of retinal diseases (32,33). The choroid may be affected by diabetes even before clinical signs of DR are present in the CFP, especially in patients with some degree of DKD, and the choriocapillaris may be preserved in the early stages (34). Microaneurysms, venous beading, vascular leakage, and nonperfused areas can be visualized by fundus fluorescein angiography, but this method is invasive, time-consuming, and not quantitative. Although OCT cannot determine blood flow, optical coherence angiography (OCTA) is a rapid, noninvasive, and high-resolution ocular fundus examination that can simultaneously perform tomographic scans and obtain blood flow information. Studies have suggested that OCTA may outperform traditional imaging techniques for the evaluation of early peripheral retinal vascular change (35).

Our study has some limitations. First, the cross-sectional design precludes the inference of causality since a temporal sequence cannot be established. Second, patients were selected from a single tertiary hospital, and the population evaluated is composed of patients with diabetes and DKD referred for retinography assessment, which limits external validity and makes it impossible to extrapolate the results to patients with diabetes in the general population. Third, GFR was estimated and not measured by the gold standard method (36), and a single urine sample was used to define DKD, which may not have ensured the exclusion of other causes of increased albuminuria, resulting in overreporting or underreporting of the true DKD prevalence in this population. In the present study, the DKD prevalence was 41.1% in patients with type 1 diabetes and 65.1% in patients with type 2 diabetes. As previous evidence suggests, differences in DKD prevalence are found all over the world and have been attributed to different methodological, social, and ethnic factors (5). In the study by Gomes et al., the prevalence of DKD in patients with type 1 diabetes was 33.7% (31). The longer diabetes duration in the patients in our cohort compared with that in the study by Gomes et al. may explain the difference in DKD prevalence between both studies. Also, the patients included in the present study were those with diabetes more difficult to control, referred for follow-up for a more complex level of care. Fourth, the definition of DKD was based on single urine and blood samples, and the definition of DKD applied, although highly accurate, was not the same as that defined by current guidelines (spot UAC > 30 mg/L or GFR < 60 mL/min/1.73 m^2^ for longer than 3 months) (19,18). Fifth, the present study was based on the evaluation of CFPs, while studies have shown that OCT can detect earlier changes in the vascular and retinal morphology, making it an important tool for the management and follow-up of retinal diseases, such as macular edema (32,33).

In conclusion, in summary, the present study showed that insulin use, longer diabetes duration, and higher SBP levels were predictors for the presence of DR in patients with type 2 diabetes and DKD. This finding suggests that patients with these characteristics should be monitored more closely for early identification of DR development. Studies with larger sample sizes are needed to identify DR predictors among patients with type 1 diabetes and DKD. The main limitations of the study include the fact that the definition of DKD adopted was not the same as that defined by current guidelines and the population evaluated was composed of patients with diabetes and DKD referred for CFP evaluation, thus the findings may not be applicable to patients with diabetes in the general population.
